# Supplementary material for: Localized Atrial Tachycardia and Dispersion Regions in Atrial Fibrillation: Evidence of Spatial Concordance
Source: J Clin Med. 2021 Jul 18;10(14):3170. doi: 10.3390/jcm10143170 (PMC8304729; doi:10.3390/jcm10143170)
Supplement: Supplementary file 1 [file jcm-10-03170-s001.zip › jcm-1288985-supplementary-conversion.pdf]

Supplemental Materials

Figure S1: Bi-atrial cumulative schematic of atrial fibrillation termination sites clearly identified in 49 patients.

Left: antero-posterior view, right: postero-anterior view. Red tags = left sided termination sites, green tags = right sided termination sites.

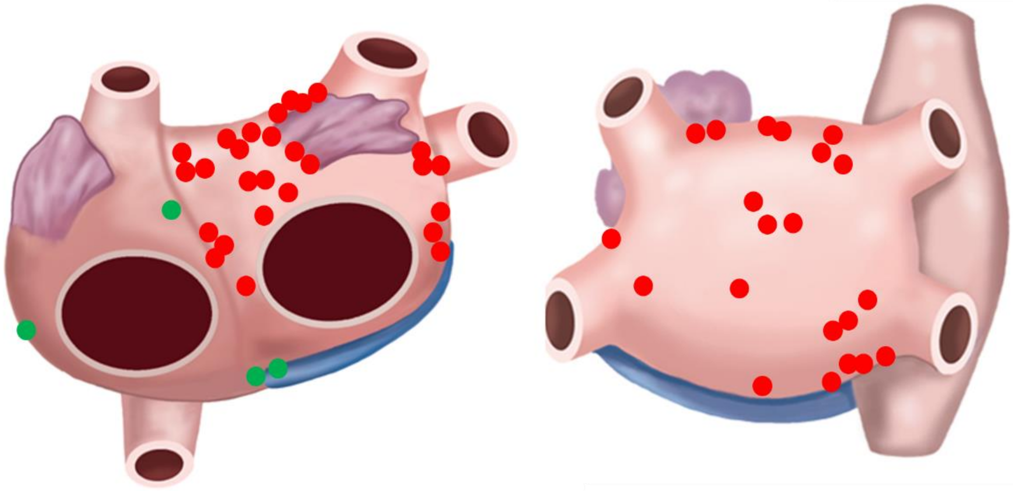

Figure S2: Bi-atrial cumulative schematic of localized atrial tachycardia termination sites in 40 patients.

Left: antero-posterior view, right: postero-anterior view. Red tags = left sided termination sites, green tags = right sided termination sites.

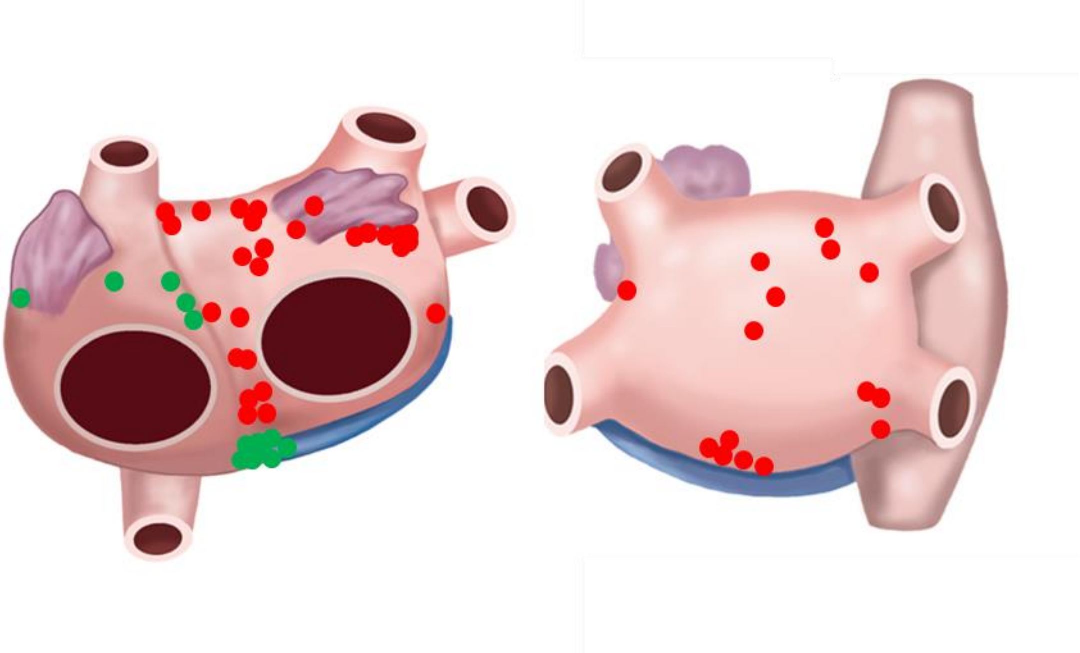

Supplemental Table S1: Arrhythmia type/mechanism/termination site for redo procedures performed between 16/11/2016 to 01/20/2021:

Patient number	Redo arrhythmia type	Arrhythmia mechanism/termination site	SInus rhythm conversion by ablation
Patient #1	AT	Peri-tricuspid flutter, localized ATs: ostium of the coronary sinus, left septum	yes
Patient #5	AF	Localized ATs: right septum, left septum	yes
Patient #6	AF	Localized ATs: left anterior wall, left atrium roof	yes
Patient #9 (redo 1)	AF	Localized ATs: left appendage anterior root, left septum, left atrium posterior wall	yes
Patient #9 (redo 2)	AT	Localized ATs: left superior PV, ostium of the coronary sinu. Peri-mitral flutter	yes
Patient #11	AT	Localized ATs: ridge, left atrium roof	yes
Patient #14 (redo 1)	AT	Localized ATs: ridge, right superior PV	yes
Patient #14 (redo 2)	AT	Localized ATs: left atrium anterior wall. Peri-tricuspid flutter,	yes
Patient #15	AT	Localized ATs: left atrium anterior wall ,left septum, right atrium lateral wall	yes
Patient #16	AT	Roof dependent flutter. Localized ATs: left appendage anterior root. Peri-mitral flutter	yes
Patient #18	AT	Peri-mitral flutter; Localized ATs: left inferior PV, left atrium anterior wall	yes
Patient #19 (redo 1)	AT	Localized ATs: left septum. Roof-dependent flutter. Localized ATs: left atrium posterior wall	yes
Patient #19 (redo 2)	AT	Localized ATs: left septum, left atrium posterior wall, right septum	yes
Patient #19 (redo 3)	AF/AT	Localized ATs: left atrium posterior wall, right onferior PV	yes
Patient #23	AT	Localized ATs: anterior mitral anulus, ridge	yes
Patient #24	AT	Peri-tricuspid flutter; Localized ATs: left septum, left appendage anterior root	yes
Patient #26	AT	Localized ATs: Ridge. Peri-tricuspid flutter	yes
Patient #29	AT	Localized ATs: Left atrium floor, left septum, ridge, right atrium lateral wall, right appendage, left atrium roof	yes
Patient #32	AF/AT	Right atrium. Peri-tricuspid flutter	yes
Patient #33 (redo 1)	AT	Peri-mitral flutter, roof-dependent flutter	yes
Patient #33 (redo 2)	AF	Localized ATs: Left PV antrum	yes
Patient #33 (redo 1)	AF	Localized ATs: left septum, PV antra	yes
Patient #34 (redo 2)	AT	Localized ATs: Left appendage anterior root, left atrium floor	yes
Patient #35 (redo 1)	AT	Localized ATs: left atrium posterior wall, right atrium lateral wall. Peri-tricuspid flutter	yes
Patient #36	AT	Roof-dependent flutter, peri-mitral flutter; Localized ATs: Left septum. Peri-tricuspid flutter.	yes
Patient #37	AT	Localized ATs: left atrium anterior wall, ridge	yes
Patient #38 (redo 1)	AT	Localized ATs: proximal coronary sinus	yes
Patient #38 (redo 2)	AT	Roof-dependent flutter, peri-mitral flutter	yes
Patient #40 (redo 1)	AT	Roof-dependent flutter. Localized ATs: ridge, distal coronary sinus	yes
Patient #40 (redo 2)	AT	Roof-dependent flutter, peri-mitral flutter. Localized ATs: proximal coronary sinus	yes
Patient #41 (redo 1)	AT	Localized ATs: left atrium posteror wall	yes
Patient #41 (redo 2)	AT	Localized ATs: proximal coronary sinus, right atrium lateral wall, right septum	yes
Patient #45 (redo 1)	AT	Localized ATs: left septum	yes
Patient #45 (redo 2)	AT	Roof-dependent flutter. Localized ATs: right PV carina	yes
Patient #48	AT	Localized ATs: ostium of the coronary sinus	yes
Patient #51	AT	Roof-dependent flutter. Localized ATs: right inferior PV. Peri-mitral flutter	yes
Patient #52 (redo 1)	AT	Localized ATs: Ridge, left appendage (not ablated)	no
Patient #52 (redo 2)	AF	Roof-dependent flutter. Localized ATs: ostium of the coronary sinus	yes
Patient #55 (redo 1)	AF/AT	Left atrium postrior wall, Roof-dependent flutter. Localized ATs: ridge	yes
Patient #55 (redo 2)	AF	Localized ATs: left atrium postrior wal, left septum	yes
Patient #56	AF	Localized ATs: right appendage, right septum	yes
Patient #57 (redo 1)	AF	Localized ATs: left atrium posterior wall, right atrium lateral wall. Peri-mitral flutter	yes
Patient #57 (redo 2)	AF	Localized ATs: left atrium posterior wall, left inferior PV	yes
Patient #59 (redo 1)	AT	Roof-dependent flutter, peri-mitral flutter	yes
Patient #59 (redo 2)	AT	Localized ATs: left atrium anterior	yes
Patient #61 (redo 1)	AF	Localized ATs: roof and mitral isthmus	yes
Patient #61 (redo 2)	AF	Localized ATs: roof	yes
Patient #63 (redo 1)	AF	Localized ATs: right septum	yes
Patient #63 (redo 2)	AF	No termination	no
Patient #68 (redo 1)	AT	Localized ATs: left septum. Peri-tricuspid flutter	yes
Patient #68 (redo 2)	AT	Localized ATs: proximal coronary sinus. Roof-dependent flutter	yes
Patient #70	AF	Localized ATs: left inferior PV	yes
Patient #71 (redo 1)	AT	Roof-dependent flutter, peri-mitral flutter	yes
Patient #71 (redo 2)	AT	Localized ATs: right appendage	yes
Patient #71 (redo 3)	AT	Peri-tricuspid flutter	yes
